# Supplementary material for: Safety Following COVID-19 Booster Vaccine with BNT162b2 Compared to mRNA-1273 in Solid Cancer Patients Previously Vaccinated with ChAdOx1 or CoronaVac
Source: Vaccines (Basel). 2023 Feb 3;11(2):356. doi: 10.3390/vaccines11020356 (PMC9965854; doi:10.3390/vaccines11020356)
Supplement: Supplementary file 1 [file vaccines-11-00356-s001.zip › vaccines-2186032-supplementary.pdf]

Supplementary material S1: Questionnaire

Volunteer number___________________ Date of vaccination _ _/_ _/ _ _ _ _ (Date/Month/Year)

Please placing check mark √ in the box ¨ that corresponds to your symptom after vaccination

1.Local reactions

	Date of onset							
	Day 1	Day 2	Day 3	Day 4	Day 5	Day 6	Day 7	After Day 7
**Pain at the injection site**	☐ No symptom<br>☐ Mild<br>☐ Moderate<br>☐ Severe<br>☐ ER visit/ hospitalization	☐ No symptom<br>☐ Mild<br>☐ Moderate<br>☐ Severe<br>☐ ER visit/ hospitalization	☐ No symptom<br>☐ Mild<br>☐ Moderate<br>☐ Severe<br>☐ ER visit/ hospitalization	☐ No symptom<br>☐ Mild<br>☐ Moderate<br>☐ Severe<br>☐ ER visit/ hospitalization	☐ No symptom<br>☐ Mild<br>☐ Moderate<br>☐ Severe<br>☐ ER visit/ hospitalization	☐ No symptom<br>☐ Mild<br>☐ Moderate<br>☐ Severe<br>☐ ER visit/ hospitalization	☐ No symptom<br>☐ Mild<br>☐ Moderate<br>☐ Severe<br>☐ ER visit/ hospitalization	☐ Absence<br>☐ Presence<br>If presence, date of symptom disappearance was _ _/_ _/_ _
**Swelling at the injection site**	☐ No symptom<br>☐ Mild (2.5 - 5 cm.)<br>☐ Moderate (5.1 – 10 cm.)<br>☐ Severe (> 10 cm.)<br>☐ Necrosis	☐ No symptom<br>☐ Mild (2.5 - 5 cm.)<br>☐ Moderate (5.1 – 10 cm.)<br>☐ Severe (> 10 cm.)<br>☐ Necrosis	☐ No symptom<br>☐ Mild (2.5 - 5 cm.)<br>☐ Moderate (5.1 – 10 cm.)<br>☐ Severe (> 10 cm.)<br>☐ Necrosis	☐ No symptom<br>☐ Mild (2.5 - 5 cm.)<br>☐ Moderate (5.1 – 10 cm.)<br>☐ Severe (> 10 cm.)<br>☐ Necrosis	☐ No symptom<br>☐ Mild (2.5 - 5 cm.)<br>☐ Moderate (5.1 – 10 cm.)<br>☐ Severe (> 10 cm.)<br>☐ Necrosis	☐ No symptom<br>☐ Mild (2.5 - 5 cm.)<br>☐ Moderate (5.1 – 10 cm.)<br>☐ Severe (> 10 cm.)<br>☐ Necrosis	☐ No symptom<br>☐ Mild (2.5 - 5 cm.)<br>☐ Moderate (5.1 – 10 cm.)<br>☐ Severe (> 10 cm.)<br>☐ Necrosis	☐ Absence<br>☐ Presence<br>If presence, date of symptom disappearance was _ _/_ _/_ _
**Erythema/Redness at the injection site**	☐ No symptom<br>☐ Mild (2.5 - 5 cm.)<br>☐ Moderate (5.1 – 10 cm.)<br>☐ Severe (> 10 cm.)<br>☐ Necrosis/ exfoliative dermatitis<br>.	☐ No symptom<br>☐ Mild (2.5 - 5 cm.)<br>☐ Moderate (5.1 – 10 cm.)<br>☐ Severe (> 10 cm.)<br>☐ Necrosis/ exfoliative dermatitis	☐ No symptom<br>☐ Mild (2.5 - 5 cm.)<br>☐ Moderate (5.1 – 10 cm.)<br>☐ Severe (> 10 cm.)<br>☐ Necrosis/ exfoliative dermatitis	☐ No symptom<br>☐ Mild (2.5 - 5 cm.)<br>☐ Moderate (5.1 – 10 cm.)<br>☐ Severe (> 10 cm.)<br>☐ Necrosis/ exfoliative dermatitis	☐ No symptom<br>☐ Mild (2.5 - 5 cm.)<br>☐ Moderate (5.1 – 10 cm.)<br>☐ Severe (> 10 cm.)<br>☐ Necrosis/ exfoliative dermatitis	☐ No symptom<br>☐ Mild (2.5 - 5 cm.)<br>☐ Moderate (5.1 – 10 cm.)<br>☐ Severe (> 10 cm.)<br>☐ Necrosis/ exfoliative dermatitis	☐ No symptom<br>☐ Mild (2.5 - 5 cm.)<br>☐ Moderate (5.1 – 10 cm.)<br>☐ Severe (> 10 cm.)<br>☐ Necrosis/ exfoliative dermatitis	☐ Absence<br>☐ Presence<br>If presence, date of symptom disappearance was _ _/_ _/_ _

2.Systemic reactions

	Date of onset							
	Day 1	Day 2	Day 3	Day 4	Day 5	Day 6	Day 7	After Day 7
Fever	☐ No<br>☐ Mild<br>(38 – 38.4 ºc)<br>☐ Moderate<br>(38.5 – 38.9 ºc)<br>☐ Severe<br>(39 – 40 ºc)<br>☐ life threatening<br>(> 40 ºc)	☐ No<br>☐ Mild<br>(38 – 38.4 ºc)<br>☐ Moderate<br>(38.5 – 38.9 ºc)<br>☐ Severe<br>(39 – 40 ºc)<br>☐ life threatening<br>(> 40 ºc)	☐ No<br>☐ Mild<br>(38 – 38.4 ºc)<br>☐ Moderate<br>(38.5 – 38.9 ºc)<br>☐ Severe<br>(39 – 40 ºc)<br>☐ life threatening<br>(> 40 ºc)	☐ No<br>☐ Mild<br>(38 – 38.4 ºc)<br>☐ Moderate<br>(38.5 – 38.9 ºc)<br>☐ Severe<br>(39 – 40 ºc)<br>☐ life threatening<br>(> 40 ºc)	☐ No<br>☐ Mild<br>(38 – 38.4 ºc)<br>☐ Moderate<br>(38.5 – 38.9 ºc)<br>☐ Severe<br>(39 – 40 ºc)<br>☐ life threatening<br>(> 40 ºc)	☐ No<br>☐ Mild<br>(38 – 38.4 ºc)<br>☐ Moderate<br>(38.5 – 38.9 ºc)<br>☐ Severe<br>(39 – 40 ºc)<br>☐ life threatening<br>(> 40 ºc)	☐ No<br>☐ Mild<br>(38 – 38.4 ºc)<br>☐ Moderate<br>(38.5 – 38.9 ºc)<br>☐ Severe<br>(39 – 40 ºc)<br>☐ life threatening<br>(> 40 ºc)	☐ Absence<br>☐ Presence<br>If presence, date of symptom disappearance was _ _/_ _/_ _
Headache	☐ No symptom<br>☐ Mild<br>(No interference with activity)<br>☐ Moderate<br>(Repeated use of non-narcotic pain reliever > 24 hours or some interference with activity)<br>☐ Severe<br>(Any use of narcotic pain reliever or prevents daily activity)<br>☐ life threatening<br>(ER visit/ hospitalization)	☐ No symptom<br>☐ Mild<br>(No interference with activity)<br>☐ Moderate<br>(Repeated use of non-narcotic pain reliever > 24 hours or some interference with activity)<br>☐ Severe<br>(Any use of narcotic pain reliever or prevents daily activity)<br>☐ life threatening<br>(ER visit/ hospitalization)	☐ No symptom<br>☐ Mild<br>(No interference with activity)<br>☐ Moderate<br>(Repeated use of non-narcotic pain reliever > 24 hours or some interference with activity)<br>☐ Severe<br>(Any use of narcotic pain reliever or prevents daily activity)<br>☐ life threatening<br>(ER visit/ hospitalization)	☐ No symptom<br>☐ Mild<br>(No interference with activity)<br>☐ Moderate<br>(Repeated use of non-narcotic pain reliever > 24 hours or some interference with activity)<br>☐ Severe<br>(Any use of narcotic pain reliever or prevents daily activity)<br>☐ life threatening<br>(ER visit/ hospitalization)	☐ No symptom<br>☐ Mild<br>(No interference with activity)<br>☐ Moderate<br>(Repeated use of non-narcotic pain reliever > 24 hours or some interference with activity)<br>☐ Severe<br>(Any use of narcotic pain reliever or prevents daily activity)<br>☐ life threatening<br>(ER visit/ hospitalization)	☐ No symptom<br>☐ Mild<br>(No interference with activity)<br>☐ Moderate<br>(Repeated use of non-narcotic pain reliever > 24 hours or some interference with activity)<br>☐ Severe<br>(Any use of narcotic pain reliever or prevents daily activity)<br>☐ life threatening<br>(ER visit/ hospitalization)	☐ No symptom<br>☐ Mild<br>(No interference with activity)<br>☐ Moderate<br>(Repeated use of non-narcotic pain reliever > 24 hours or some interference with activity)<br>☐ Severe<br>(Any use of narcotic pain reliever or prevents daily activity)<br>☐ life threatening<br>(ER visit/ hospitalization)	☐ Absence<br>☐ Presence<br>If presence, date of symptom disappearance was _ _/_ _/_ _
Myalgia	☐ No symptom<br>☐ Mild<br>(No interference with activity)<br>☐ Moderate<br>(Repeated use of non-narcotic pain reliever > 24 hours or some interference with activity)<br>☐ Severe<br>(Any use of narcotic pain reliever or prevents daily activity)<br>☐ life threatening	☐ No symptom<br>☐ Mild<br>(No interference with activity)<br>☐ Moderate<br>(Repeated use of non-narcotic pain reliever > 24 hours or some interference with activity)<br>☐ Severe<br>(Any use of narcotic pain reliever or prevents daily activity)<br>☐ life threatening	☐ No symptom<br>☐ Mild<br>(No interference with activity)<br>☐ Moderate<br>(Repeated use of non-narcotic pain reliever > 24 hours or some interference with activity)<br>☐ Severe<br>(Any use of narcotic pain reliever or prevents daily activity)<br>☐ life threatening	☐ No symptom<br>☐ Mild<br>(No interference with activity)<br>☐ Moderate<br>(Repeated use of non-narcotic pain reliever > 24 hours or some interference with activity)<br>☐ Severe<br>(Any use of narcotic pain reliever or prevents daily activity)<br>☐ life threatening	☐ No symptom<br>☐ Mild<br>(No interference with activity)<br>☐ Moderate<br>(Repeated use of non-narcotic pain reliever > 24 hours or some interference with activity)<br>☐ Severe<br>(Any use of narcotic pain reliever or prevents daily activity)<br>☐ life threatening	☐ No symptom<br>☐ Mild<br>(No interference with activity)<br>☐ Moderate<br>(Repeated use of non-narcotic pain reliever > 24 hours or some interference with activity)<br>☐ Severe<br>(Any use of narcotic pain reliever or prevents daily activity)<br>☐ life threatening	☐ No symptom<br>☐ Mild<br>(No interference with activity)<br>☐ Moderate<br>(Repeated use of non-narcotic pain reliever > 24 hours or some interference with activity)<br>☐ Severe<br>(Any use of narcotic pain reliever or prevents daily activity)<br>☐ life threatening	☐ Absence<br>☐ Presence<br>If presence, date of symptom disappearance was _ _/_ _/_ _

Date of onset								
	Day 1	Day 2	Day 3	Day 4	Day 5	Day 6	Day 7	After Day 7
	(ER visit/ hospitalization)	(ER visit/ hospitalization)	(ER visit/ hospitalization)	(ER visit/ hospitalization)	(ER visit/ hospitalization)	(ER visit/ hospitalization)	(ER visit/ hospitalization)	
Fatigue	☐ No symptom<br>☐ Mild<br>(No interference with activity)<br>☐ Moderate<br>(Some interference with activity)<br>☐ Severe<br>(Prevents daily activity)<br>☐ life threatening<br>(ER visit/ hospitalization)	☐ No symptom<br>☐ Mild<br>(No interference with activity)<br>☐ Moderate<br>(Some interference with activity)<br>☐ Severe<br>(Prevents daily activity)<br>☐ life threatening<br>(ER visit/ hospitalization)	☐ No symptom<br>☐ Mild<br>(No interference with activity)<br>☐ Moderate<br>(Some interference with activity)<br>☐ Severe<br>(Prevents daily activity)<br>☐ life threatening<br>(ER visit/ hospitalization)	☐ No symptom<br>☐ Mild<br>(No interference with activity)<br>☐ Moderate<br>(Some interference with activity)<br>☐ Severe<br>(Prevents daily activity)<br>☐ life threatening<br>(ER visit/ hospitalization)	☐ No symptom<br>☐ Mild<br>(No interference with activity)<br>☐ Moderate<br>(Some interference with activity)<br>☐ Severe<br>(Prevents daily activity)<br>☐ life threatening<br>(ER visit/ hospitalization)	☐ No symptom<br>☐ Mild<br>(No interference with activity)<br>☐ Moderate<br>(Some interference with activity)<br>☐ Severe<br>(Prevents daily activity)<br>☐ life threatening<br>(ER visit/ hospitalization)	☐ No symptom<br>☐ Mild<br>(No interference with activity)<br>☐ Moderate<br>(Some interference with activity)<br>☐ Severe<br>(Prevents daily activity)<br>☐ life threatening<br>(ER visit/ hospitalization)	☐ Absence<br>☐ Presence<br>If presence, date of symptom disappearance was _ _/_ _/_ _
Nausea and vomiting	☐ No symptom<br>☐ Mild<br>(1-2 episodes/24 hr, no interference with activity)<br>☐ Moderate<br>(>2 episodes/24 hr, some interference with activity)<br>☐ Severe<br>(Prevents daily activity, requires IV hydration)<br>☐ Life threatening<br>(Hypotensive shock, ER visit/ hospitalization)	☐ No symptom<br>☐ Mild<br>(1-2 episodes/24 hr, no interference with activity)<br>☐ Moderate<br>(>2 episodes/24 hr, some interference with activity)<br>☐ Severe<br>(Prevents daily activity, requires IV hydration)<br>☐ Life threatening<br>(Hypotensive shock, ER visit/ hospitalization)	☐ No symptom<br>☐ Mild<br>(1-2 episodes/24 hr, no interference with activity)<br>☐ Moderate<br>(>2 episodes/24 hr, some interference with activity)<br>☐ Severe<br>(Prevents daily activity, requires IV hydration)<br>☐ Life threatening<br>(Hypotensive shock, ER visit/ hospitalization)	☐ No symptom<br>☐ Mild<br>(1-2 episodes/24 hr, no interference with activity)<br>☐ Moderate<br>(>2 episodes/24 hr, some interference with activity)<br>☐ Severe<br>(Prevents daily activity, requires IV hydration)<br>☐ Life threatening<br>(Hypotensive shock, ER visit/ hospitalization)	☐ No symptom<br>☐ Mild<br>(1-2 episodes/24 hr, no interference with activity)<br>☐ Moderate<br>(>2 episodes/24 hr, some interference with activity)<br>☐ Severe<br>(Prevents daily activity, requires IV hydration)<br>☐ Life threatening<br>(Hypotensive shock, ER visit/ hospitalization)	☐ No symptom<br>☐ Mild<br>(1-2 episodes/24 hr, no interference with activity)<br>☐ Moderate<br>(>2 episodes/24 hr, some interference with activity)<br>☐ Severe<br>(Prevents daily activity, requires IV hydration)<br>☐ Life threatening<br>(Hypotensive shock, ER visit/ hospitalization)	☐ No symptom<br>☐ Mild<br>(1-2 episodes/24 hr, no interference with activity)<br>☐ Moderate<br>(>2 episodes/24 hr, some interference with activity)<br>☐ Severe<br>(Prevents daily activity, requires IV hydration)<br>☐ Life threatening<br>(Hypotensive shock, ER visit/ hospitalization)	☐ Absence<br>☐ Presence<br>If presence, date of symptom disappearance was _ _/_ _/_ _
Diarrhea	☐ No symptom<br>☐ Mild<br>(2-3 loose stools/24 hr)<br>☐ Moderate<br>(4-5 loose stools/24 hr)<br>☐ Severe<br>(> 6 loose stools/24 hr, requires IV hydration)<br>☐ Life threatening<br>(Hypotensive shock, ER visit/ hospitalization)	☐ No symptom<br>☐ Mild (2-3 loose stools/24 hr)<br>☐ Moderate (4-5 loose stools/24 hr)<br>☐ Severe<br>(> 6 loose stools/24 hr, requires IV hydration)<br>☐ Life threatening<br>(Hypotensive shock, ER visit/ hospitalization)	☐ No symptom<br>☐ Mild (2-3 loose stools/24 hr)<br>☐ Moderate (4-5 loose stools/24 hr)<br>☐ Severe<br>(> 6 loose stools/24 hr, requires IV hydration)<br>☐ Life threatening<br>(Hypotensive shock, ER visit/ hospitalization)	☐ No symptom<br>☐ Mild (2-3 loose stools/24 hr)<br>☐ Moderate (4-5 loose stools/24 hr)<br>☐ Severe<br>(> 6 loose stools/24 hr, requires IV hydration)<br>☐ Life threatening<br>(Hypotensive shock, ER visit/ hospitalization)	☐ No symptom<br>☐ Mild (2-3 loose stools/24 hr)<br>☐ Moderate (4-5 loose stools/24 hr)<br>☐ Severe<br>(> 6 loose stools/24 hr, requires IV hydration)<br>☐ Life threatening<br>(Hypotensive shock, ER visit/ hospitalization)	☐ No symptom<br>☐ Mild (2-3 loose stools/24 hr)<br>☐ Moderate (4-5 loose stools/24 hr)<br>☐ Severe<br>(> 6 loose stools/24 hr, requires IV hydration)<br>☐ Life threatening<br>(Hypotensive shock, ER visit/ hospitalization)	☐ No symptom<br>☐ Mild (2-3 loose stools/24 hr)<br>☐ Moderate (4-5 loose stools/24 hr)<br>☐ Severe<br>(> 6 loose stools/24 hr, requires IV hydration)<br>☐ Life threatening<br>(Hypotensive shock, ER visit/ hospitalization)	☐ Absence<br>☐ Presence<br>If presence, date of symptom disappearance was _ _/_ _/_ _

Date of onset								
	Day 1	Day 2	Day 3	Day 4	Day 5	Day 6	Day 7	After Day 7
Dizziness or Vertigo	☐ No symptom<br>☐ Mild<br>(No interference with activity)<br>☐ Moderate<br>(Some interference with activity)<br>☐ Severe<br>(Prevents daily activity)<br>☐ life threatening<br>(ER visit/ hospitalization)	☐ No symptom<br>☐ Mild<br>(No interference with activity)<br>☐ Moderate<br>(Some interference with activity)<br>☐ Severe<br>(Prevents daily activity)<br>☐ life threatening<br>(ER visit/ hospitalization)	☐ No symptom<br>☐ Mild<br>(No interference with activity)<br>☐ Moderate<br>(Some interference with activity)<br>☐ Severe<br>(Prevents daily activity)<br>☐ life threatening<br>(ER visit/ hospitalization)	☐ No symptom<br>☐ Mild<br>(No interference with activity)<br>☐ Moderate<br>(Some interference with activity)<br>☐ Severe<br>(Prevents daily activity)<br>☐ life threatening<br>(ER visit/ hospitalization)	☐ No symptom<br>☐ Mild<br>(No interference with activity)<br>☐ Moderate<br>(Some interference with activity)<br>☐ Severe<br>(Prevents daily activity)<br>☐ life threatening<br>(ER visit/ hospitalization)	☐ No symptom<br>☐ Mild<br>(No interference with activity)<br>☐ Moderate<br>(Some interference with activity)<br>☐ Severe<br>(Prevents daily activity)<br>☐ life threatening<br>(ER visit/ hospitalization)	☐ No symptom<br>☐ Mild<br>(No interference with activity)<br>☐ Moderate<br>(Some interference with activity)<br>☐ Severe<br>(Prevents daily activity)<br>☐ life threatening<br>(ER visit/ hospitalization)	☐ Absence<br>☐ Presence<br>If presence, date of symptom disappearance was _ _/_ _/_ _
Joint pain	☐ No symptom<br>☐ Mild<br>(No interference with activity)<br>☐ Moderate<br>(Repeated use of non-narcotic pain reliever > 24 hours or some interference with activity)<br>☐ Severe<br>(Any use of narcotic pain reliever or prevents daily activity)<br>☐ life threatening<br>(ER visit/ hospitalization)	☐ No symptom<br>☐ Mild<br>(No interference with activity)<br>☐ Moderate<br>(Repeated use of non-narcotic pain reliever > 24 hours or some interference with activity)<br>☐ Severe<br>(Any use of narcotic pain reliever or prevents daily activity)<br>☐ life threatening<br>(ER visit/ hospitalization)	☐ No symptom<br>☐ Mild<br>(No interference with activity)<br>☐ Moderate<br>(Repeated use of non-narcotic pain reliever > 24 hours or some interference with activity)<br>☐ Severe<br>(Any use of narcotic pain reliever or prevents daily activity)<br>☐ life threatening<br>(ER visit/ hospitalization)	☐ No symptom<br>☐ Mild<br>(No interference with activity)<br>☐ Moderate<br>(Repeated use of non-narcotic pain reliever > 24 hours or some interference with activity)<br>☐ Severe<br>(Any use of narcotic pain reliever or prevents daily activity)<br>☐ life threatening<br>(ER visit/ hospitalization)	☐ No symptom<br>☐ Mild<br>(No interference with activity)<br>☐ Moderate<br>(Repeated use of non-narcotic pain reliever > 24 hours or some interference with activity)<br>☐ Severe<br>(Any use of narcotic pain reliever or prevents daily activity)<br>☐ life threatening<br>(ER visit/ hospitalization)	☐ No symptom<br>☐ Mild<br>(No interference with activity)<br>☐ Moderate<br>(Repeated use of non-narcotic pain reliever > 24 hours or some interference with activity)<br>☐ Severe<br>(Any use of narcotic pain reliever or prevents daily activity)<br>☐ life threatening<br>(ER visit/ hospitalization)	☐ No symptom<br>☐ Mild<br>(No interference with activity)<br>☐ Moderate<br>(Repeated use of non-narcotic pain reliever > 24 hours or some interference with activity)<br>☐ Severe<br>(Any use of narcotic pain reliever or prevents daily activity)<br>☐ life threatening<br>(ER visit/ hospitalization)	☐ Absence<br>☐ Presence<br>If presence, date of symptom disappearance was _ _/_ _/_ _
Back pain	☐ No symptom<br>☐ Mild<br>(No interference with activity)<br>☐ Moderate<br>(Repeated use of non-narcotic pain reliever > 24 hours or some interference with activity)<br>☐ Severe<br>(Any use of narcotic pain reliever or prevents daily activity)<br>☐ life threatening	☐ No symptom<br>☐ Mild<br>(No interference with activity)<br>☐ Moderate<br>(Repeated use of non-narcotic pain reliever > 24 hours or some interference with activity)<br>☐ Severe<br>(Any use of narcotic pain reliever or prevents daily activity)<br>☐ life threatening	☐ No symptom<br>☐ Mild<br>(No interference with activity)<br>☐ Moderate<br>(Repeated use of non-narcotic pain reliever > 24 hours or some interference with activity)<br>☐ Severe<br>(Any use of narcotic pain reliever or prevents daily activity)<br>☐ life threatening	☐ No symptom<br>☐ Mild<br>(No interference with activity)<br>☐ Moderate<br>(Repeated use of non-narcotic pain reliever > 24 hours or some interference with activity)<br>☐ Severe<br>(Any use of narcotic pain reliever or prevents daily activity)<br>☐ life threatening	☐ No symptom<br>☐ Mild<br>(No interference with activity)<br>☐ Moderate<br>(Repeated use of non-narcotic pain reliever > 24 hours or some interference with activity)<br>☐ Severe<br>(Any use of narcotic pain reliever or prevents daily activity)<br>☐ life threatening	☐ No symptom<br>☐ Mild<br>(No interference with activity)<br>☐ Moderate<br>(Repeated use of non-narcotic pain reliever > 24 hours or some interference with activity)<br>☐ Severe<br>(Any use of narcotic pain reliever or prevents daily activity)<br>☐ life threatening	☐ No symptom<br>☐ Mild<br>(No interference with activity)<br>☐ Moderate<br>(Repeated use of non-narcotic pain reliever > 24 hours or some interference with activity)<br>☐ Severe<br>(Any use of narcotic pain reliever or prevents daily activity)<br>☐ life threatening	☐ Absence<br>☐ Presence<br>If presence, date of symptom disappearance was _ _/_ _/_ _

Date of onset								
	Day 1	Day 2	Day 3	Day 4	Day 5	Day 6	Day 7	After Day 7
	(ER visit/ hospitalization)	(ER visit/ hospitalization)	(ER visit/ hospitalization)	(ER visit/ hospitalization)	(ER visit/ hospitalization)	(ER visit/ hospitalization)	(ER visit/ hospitalization)	
Lump/ swollen at axillary /other lymph nodes	☐ No symptom<br>☐ Mild<br>(No interference with activity)<br>☐ Moderate<br>(Some interference with activity)<br>☐ Severe<br>(Prevents daily activity)<br>☐ Life threatening<br>(ER visit/ hospitalization)	☐ No symptom<br>☐ Mild<br>(No interference with activity)<br>☐ Moderate<br>(Some interference with activity)<br>☐ Severe<br>(Prevents daily activity)<br>☐ Life threatening<br>(ER visit/ hospitalization)	☐ No symptom<br>☐ Mild<br>(No interference with activity)<br>☐ Moderate<br>(Some interference with activity)<br>☐ Severe<br>(Prevents daily activity)<br>☐ Life threatening<br>(ER visit/ hospitalization)	☐ No symptom<br>☐ Mild<br>(No interference with activity)<br>☐ Moderate<br>(Some interference with activity)<br>☐ Severe<br>(Prevents daily activity)<br>☐ Life threatening<br>(ER visit/ hospitalization)	☐ No symptom<br>☐ Mild<br>(No interference with activity)<br>☐ Moderate<br>(Some interference with activity)<br>☐ Severe<br>(Prevents daily activity)<br>☐ Life threatening<br>(ER visit/ hospitalization)	☐ No symptom<br>☐ Mild<br>(No interference with activity)<br>☐ Moderate<br>(Some interference with activity)<br>☐ Severe<br>(Prevents daily activity)<br>☐ Life threatening<br>(ER visit/ hospitalization)	☐ No symptom<br>☐ Mild<br>(No interference with activity)<br>☐ Moderate<br>(Some interference with activity)<br>☐ Severe<br>(Prevents daily activity)<br>☐ Life threatening<br>(ER visit/ hospitalization)	☐ Absence<br>☐ Presence<br>If presence, date of symptom disappearance was _ _/_ _/_ _
Other symptom specify<br>………..<br>………..<br>………..	☐ No symptom<br>☐ Mild<br>(No interference with activity)<br>☐ Moderate<br>(Some interference with activity)<br>☐ Severe<br>(Prevents daily activity)<br>☐ Life threatening<br>(ER visit/ hospitalization)	☐ No symptom<br>☐ Mild<br>(No interference with activity)<br>☐ Moderate<br>(Some interference with activity)<br>☐ Severe<br>(Prevents daily activity)<br>☐ Life threatening<br>(ER visit/ hospitalization)	☐ No symptom<br>☐ Mild<br>(No interference with activity)<br>☐ Moderate<br>(Some interference with activity)<br>☐ Severe<br>(Prevents daily activity)<br>☐ Life threatening<br>(ER visit/ hospitalization)	☐ No symptom<br>☐ Mild<br>(No interference with activity)<br>☐ Moderate<br>(Some interference with activity)<br>☐ Severe<br>(Prevents daily activity)<br>☐ Life threatening<br>(ER visit/ hospitalization)	☐ No symptom<br>☐ Mild<br>(No interference with activity)<br>☐ Moderate<br>(Some interference with activity)<br>☐ Severe<br>(Prevents daily activity)<br>☐ Life threatening<br>(ER visit/ hospitalization)	☐ No symptom<br>☐ Mild<br>(No interference with activity)<br>☐ Moderate<br>(Some interference with activity)<br>☐ Severe<br>(Prevents daily activity)<br>☐ Life threatening<br>(ER visit/ hospitalization)	☐ No symptom<br>☐ Mild<br>(No interference with activity)<br>☐ Moderate<br>(Some interference with activity)<br>☐ Severe<br>(Prevents daily activity)<br>☐ Life threatening<br>(ER visit/ hospitalization)	☐ Absence<br>☐ Presence<br>If presence, date of symptom disappearance was _ _/_ _/_ _
